# Immune imbalance mediates the relationship between plasma vitamin D concentration and preeclampsia in Chinese pregnant women: a case–control study

**DOI:** 10.3389/fnut.2025.1665593

**Published:** 2025-11-14

**Authors:** Huanan Chen, Yacong Bo, Yuan Cao, Jinyan Liu, Jiaqi Nie, Dandan Duan, Weifeng Dou, Xianlan Zhao, Wenjun Fu, Yi Zhang, Yanhua Liu

**Affiliations:** 1 Department of Nutrition, The First Affiliated Hospital of Zhengzhou University, Zhengzhou, China; 2 Department of Medical Record Management and Statistics, Shandong Provincial Qianfoshan Hospital and The First Affiliated Hospital of Shandong First Medical University, Jinan, China; 3 Department of Nutrition and Food Hygiene, College of Public Health, Zhengzhou University, Zhengzhou, China; 4 The Third Affiliated Hospital of Zhengzhou University, Zhengzhou, China; 5 Biotherapy Center and Cancer Center, The First Affiliated Hospital of Zhengzhou University, Zhengzhou, Henan, China; 6 Department of Clinical Nutrition, Luoyang New Area People’s Hospital, Luoyang, China; 7 Department of Obstetrics, The First Affiliated Hospital of Zhengzhou University, Zhengzhou, China; 8 School of Public Health, Zhengzhou University, Zhengzhou, Henan, China; 9 Zhongyuan Cell and Immunotherapy Laboratory, Henan Academy of Medical Sciences, Zhengzhou, China

**Keywords:** vitamin D, preeclampsia, immune imbalance, mediation effect, Chinese

## Abstract

**Background:**

Vitamin D insufficiency is associated with an increased risk of preeclampsia. However, the role of T helper cell type 1 (Th1)/Th2 and Th17/regulatory T cell (Treg) balance in this association is unclear.

**Methods:**

We conducted a case–control study to explore the mediating effects of immune balance on the relationship between the plasma vitamin D concentration and preeclampsia. This study included 373 pregnant women recruited between March 2016 and February 2019 (192 cases and 181 controls). The cytokines as well as various T cell subsets were analyzed using ELISA and flow cytometry, respectively. Spearman’s correlation was used to investigate the association between vitamin D and cytokines as well as T cell subsets and mediating analysis was performed to identify the modifying effects of immune balance on this association.

**Results:**

The Th1/Th2 and Th17/Treg ratios were negatively associated with the plasma concentration of 25-hydroxyvitamin D_2_ (25(OH)D_2_). Mediation analysis found that the Th1/Th2 as well as Th17/Treg ratios mediated the effect of 25(OH)D_2_ on the risk of preeclampsia, the mediating effects accounted for 59.59 and 40.45%, respectively.

**Conclusion:**

Our results provided preliminary evidence for a potential mediating role of the Th1/Th2 and Th17/Treg balance in the association between plasma 25(OH)D_2_ and preeclampsia. Keeping higher levels of vitamin D, especially 25(OH)D_2_ might help maintain the immune balance and decrease the risk of preeclampsia.

## Introduction

Preeclampsia, defined as the presence of new-onset hypertension and proteinuria or other end-organ damage occurring after 20 weeks of gestation, is a leading complication of pregnancy. Approximately 4–5% pregnant women worldwide are affected by preeclampsia (1–3). Preeclampsia is associated with elevated risks of eclampsia, liver rupture and stroke, as well as cardiovascular disease later in life (4, 5). Preeclampsia is also associated with fetal growth restriction and preterm birth, which increases the risks of cerebral palsy and bronchopulmonary dysplasia in offspring (6–8). Delivery and removal of the placenta is the only definitive cure for preeclampsia (9). Therefore, pregnancy is a critical window through which the future health of both the mother and offspring can be elevated. Prevention of preeclampsia is a highly important clinical goal (6, 10).

Immune imbalances, particularly between the Th1 and Th2 cell subsets as well as the Th17 and Treg cell subsets, have been identified as the main mechanism underlying preeclampsia (11, 12). In our previous study, we identified an inverse association between vitamin D and the risk of preeclampsia (13), consistent with findings from other studies (14–16). Previous studies also found that immune imbalance is independently associated with risk of preeclampsia (17), while, it remains unclear whether vitamin D is related to the immune status in preeclampsia and how vitamin D affects the preeclampsia by regulating immune balance. Thus it is imperative to clarify the mediating effects of immune imbalance between vitamin D and preeclampsia in pregnant women.

To address this knowledge gap, this case–control study focused on the epidemiological links between the plasma vitamin D concentration and immune imbalance in pregnant women and examined the potential mediating role of Th1/Th2 and Th17/Treg imbalance in the association between vitamin D and preeclampsia.

## Methods

### Participants

This case–control study was conducted between March 2016 and February 2019 at a large general hospital in central China. Pregnant women aged ≥18 years with a singleton pregnancy and a gestational age ≥28 weeks were included. Preeclampsia was diagnosed according to China’s Diagnosis and Treatment Guideline of Hypertensive Disorders in Pregnancy (2015) (18). Details of the inclusion and exclusion criteria are described elsewhere (19). This study included 373 participants (192 cases and 181 controls), and all participants provided written informed consent. The study protocol was approved by the Ethics Committee of Scientific Research and Clinical Trials at the First Affiliated Hospital of Zhengzhou University (Approval No. Scientific Research-2016-LW-34).

### Cytokine measurement

Fasting peripheral blood (FPB) was collected from participants into the sterile tubes containing EDTA on the day of delivery, which were centrifuged (4 °C, 800 g, 10 min), the plasma was then collected into tubes and stored at −80 °C. The concentrations of cytokines in plasma samples were measured using enzyme-linked immunosorbent assays (ELISAs). As described previously, interferon (IFN)-γ was used to define a Th1 response, while IL-4, IL-17A and IL-10 were used to define Th2, Th17, and Treg responses, respectively (20–23). The ELISAs were performed according to the instructions provided by the kit manufacturer. Briefly, the samples and standards were added to a microplate that was precoated with a specific mouse monoclonal antibody and incubated at temperature for 2 h. Then, wash and added to 100 μL diluted detection antibody solution to each well, incubated at temperature for 1 h. Next, wash and added to 100 μL diluted Avidin-HRP solution to each well, incubated at temperature for 30 min. Wash, and then 100 μL freshly mixed substrate solution were added to each well and the plate was incubated in the dark. Subsequently, 100 μL stop solution was added to each well to stop the reaction. Finally, the absorbance at 450 nm was read in each well using a spectrophotometer (Molecular Devices, SpectraMax iD3, United States). All the ELISA kits were purchased from BioLegend (San Diego, CA, United States). Cytokine concentrations were detected in samples from 373 participants in this study.

### Flow cytometry

T cell subsets were analyzed by flow cytometry according to the following procedure. First, FPB (the obtained time was on the day of delivery) was collected from participants into the sterile tubes containing EDTA, and then centrifuged (4 °C, 800 g, 10 min) to remove the plasma. Next, sterile normal saline was added to precipitate and mixed, which was added carefully to a sterile centrifuge tube containing lymphocyte separation solution. The mixture was subjected to density gradient centrifugation (4 °C, 800 g, 25 min). The peripheral blood mononuclear cells (PBMCs) were collected and seeded into 24-well plates at a density of 2 × 10^6^ PBMCs per mL RPMI-1640 supplemented with 10% fetal bovine plasma (Lonsera, United States) and 100 IU/mL penicillin/streptomycin. To each well, 1 μL of 1 mg/mL phorbol myristate acetate (Sigma-Aldrich), 50 ng/mL ionomycin (Sigma-Aldrich) and 5 mg/mL brefeldin A solution (BioLegend) were added, and the plate was incubated for 4–6 h at 37 °C in 5% CO_2_ and then harvested. The cells were resuspended in phosphate-buffered saline containing 0.5% bovine serum albumin and incubated with an Fc receptor blocking regent for 15 min at 4 °C. The single-cell suspensions were then incubated with fluorochrome-conjugated antibodies specific for CD3 (fluorochrome: PE-CY7), CD4 (APC-CY7), and CD25 (FITC) for 15 min at 4 °C in the dark. Next, intracellular staining using fluorochrome-conjugated antibodies specific for IL-17A (PE), IL-4 (FITC), and IFN-γ (APC) and nucleoprotein staining to detect FOXP3 (APC) were performed according to the manufacturer’s instructions. Briefly, the cells were stained with antibodies against intracellular cytokines after surface antibody staining, fixation, and permeabilization. The Th1, Th2, Th17, and Treg subsets were defined as CD3^+^ CD4^+^ IFN-γ^+^, CD3^+^ CD4^+^ IL-4^+^, CD3^+^ CD4^+^ IL-17A^+^ and CD3^+^ CD4^+^ CD25^+^ FOXP3^+^ (24–27). All of the flow cytometry antibodies were purchased from BioLegend (San Diego, CA, United States). Flow cytometry was performed using FACS Canto II flow cytometer (BD Biosciences, Franklin Lakes, NJ, United States). A total of 65 samples (including 34 cases and 31 controls) were detected T cell subsets in this study. The distribution of 65 flow cytometry samples between cases and controls showed in Supplementary Table 1. Flow Jo software was used to analyze the data.

### Variables

The information on demographic characteristics were collected using our standardized interview questionnaires. Maternal age, gestational week, parity (multiparous or primiparous), pre-pregnancy BMI, education level (high school or below, university or above) and monthly income (≤4,000 RMB or >4,000 RMB) were included in the adjusted model.

### Statistical analysis

For normally distributed data, Student’s *t* or Wilcoxon test were used to compare continuous parameters between the cases and controls, and chi-squared tests were conducted to compare differences in qualitative variables between cases and controls. Spearman’s correlation was used to analyze the correlations between vitamin D concentrations and immune parameters.

The R package “mediation” (Version 4.5.0) was used to conduct a mediation effect analysis of the potential modifying effect of immune balance on the relationship between the vitamin D concentration and the risk of preeclampsia (Y as outcome: preeclampsia or not, X as exposure: 25(OH)D/25(OH)D_2_/25(OH)D_3_, M as mediator: immune parameters). This analysis presented the direct effect of exposure on preeclampsia as well as the indirect effect bypassing the mediator. Other details of the mediation analysis were described previously (28). A two-tailed *p*-value of <0.05 was regarded as significant. All statistical analyses were performed using SPSS software, version 25.0 (SPSS Inc., Chicago, IL, United States).

## Results

The characteristics of the participants are presented in Table 1. There were no significant differences between the cases and controls in terms of maternal age, gestational week, pre-pregnant BMI or education. However, compared with controls, women with preeclampsia had a higher SBP, DBP, greater weight gain during pregnancy, a higher income level, and significantly lower serum concentrations 25(OH)D_2_, 25(OH)D_3_ and 25(OH)D. Additionally, primiparous women were more likely to suffer from preeclampsia than multiparous women.

**Table 1:** Characteristics of cases and controls.

Variables	Cases (*n* = 192)	Controls (*n* = 181)	*p*
Maternal age (years)	31.25 ± 5.48	31.78 ± 4.61	0.310
Gestational week (weeks)	33.83 ± 2.97	34.29 ± 2.27	0.092
Parity			0.006
Primiparous	78 (45.09)	54 (30.68)	
Multiparous	95 (54.91)	122 (69.32)	
SBP (mmHg)	156.77 ± 18.26	114.45 ± 11.17	<0.001
DBP (mmHg)	100.79 ± 13.15	74.14 ± 9.23	<0.001
Pre-pregnancy BMI (kg/m^2^)	23.51 ± 3.96	22.96 ± 3.47	0.170
Weight gain (kg)	16.47 ± 7.08	12.31 ± 5.21	<0.001
Monthly income			0.040
≤4,000 RMB	103 (66.88)	93 (55.69)	
>4,000 RMB	51 (33.12)	74 (44.31)	
Education level			0.144
High school or below	104 (62.65)	96 (54.86)	
University or above	62 (37.35)	79 (45.14)	
GDM			0.502
No	161 (83.85)	147 (81.22)	
Yes	31 (16.15)	34 (18.78)	
25(OH)D_2_ (ng/mL)	0.79 (0.57, 1.05)	1.06 (0.82, 1.82)	<0.001
25(OH)D_3_ (ng/mL)	9.83 (6.04, 15.90)	12.50 (8.70, 19.30)	<0.001
25(OH)D (ng/mL)	10.65 (7.25, 16.60)	14.00 (10.40, 20.70)	<0.001

DBP, diastolic blood pressure; SBP, systolic blood pressure; BMI, body mass index; GDM, gestational diabetes.

The associations between vitamin D and preeclampsia had been reported in our previous study. In brief, higher concentration of 25(OH)D was associated with a lower risk of preeclampsia, with generally similar results observed for 25(OH)D_2_ and 25(OH)D_3_ (13).

**Figure 1:**
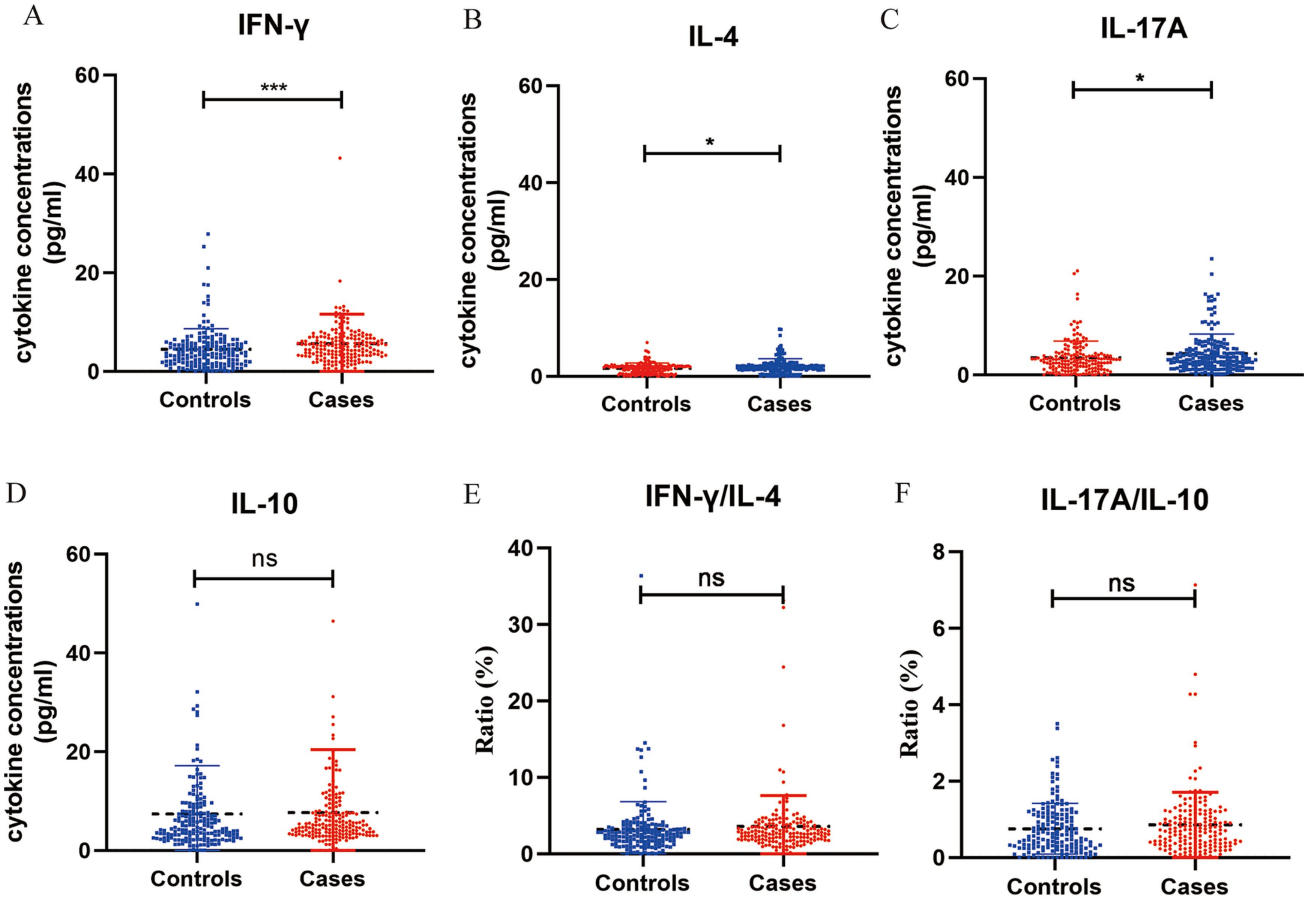
Concentration of cytokines in plasma samples from pregnant women with preeclampsia (*n* = 192) and without preeclampsia (*n* = 181). The concentration of interferon (IFN)-γ **(A)**, IL-4 **(B)**, IL-17A **(C)**, IL-10 **(D)** (pg/mL) were measured in both groups using ELISA, the ratio of IFN-γ/IL-4 **(E)** and IL-17A/IL-10 **(F)**. ^*^p < 0.05, ^**^p < 0.01, ^***^p < 0.001, and ^ns^*p* > 0.05.

The distributions of cytokines in the two groups are showed in Figure 1. Women with preeclampsia had significantly higher concentrations IFN-γ (4.94 versus 3.72 pg/mL, *p* < 0.001), IL-17A (3.31 versus 2.87 pg/mL, *p* = 0.016) and IL-4 (1.90 versus 1.74 pg/mL, *p* = 0.018) than controls, whereas no significant difference in the IL-10 concentration was observed between the groups (5.12 versus 4.47 pg/mL, *p* > 0.05). Furthermore, there were no statistically significant differences in the ratio of IFN-γ/IL-4 (3.57 versus 3.12, *p* > 0.05) and IL-17A/IL-10 (0.85 versus 0.75, *p* > 0.05) between the groups.

Table 2 showed the correlations between vitamin D and cytokines, while no significant associations were found. Supplementary Table 2 presented the correlations between cytokines and preeclampsia, and IFN-γ, IL-4 and IL-17A were significant correlated with preeclampsia. Moreover, mediating analysis suggested that cytokines did not mediate the relationship between vitamin D and preeclampsia. Detailed results were presented in Supplementary Tables 3–5.

**Table 2:** Correlations between vitamin D forms and cytokines (*n* = 373).

Cytokines	25(OH)D_2_		25(OH)D_3_		25(OH)D	
	*r*	*p*	*r*	*p*	*r*	*p*
IFN-γ	0.060	0.244	−0.025	0.626	−0.021	0.680
IL-4	−0.020	0.704	0.010	0.848	−0.010	0.849
IL-17A	−0.012	0.826	0.016	0.755	−0.005	0.918
IL-10	−0.015	0.772	−0.007	0.891	−0.017	0.740
IFN-γ/IL-4	0.017	0.754	−0.030	0.582	−0.009	0.864
IL-17A/IL-10	0.004	0.942	−0.008	0.879	−0.022	0.684

**Figure 2:**
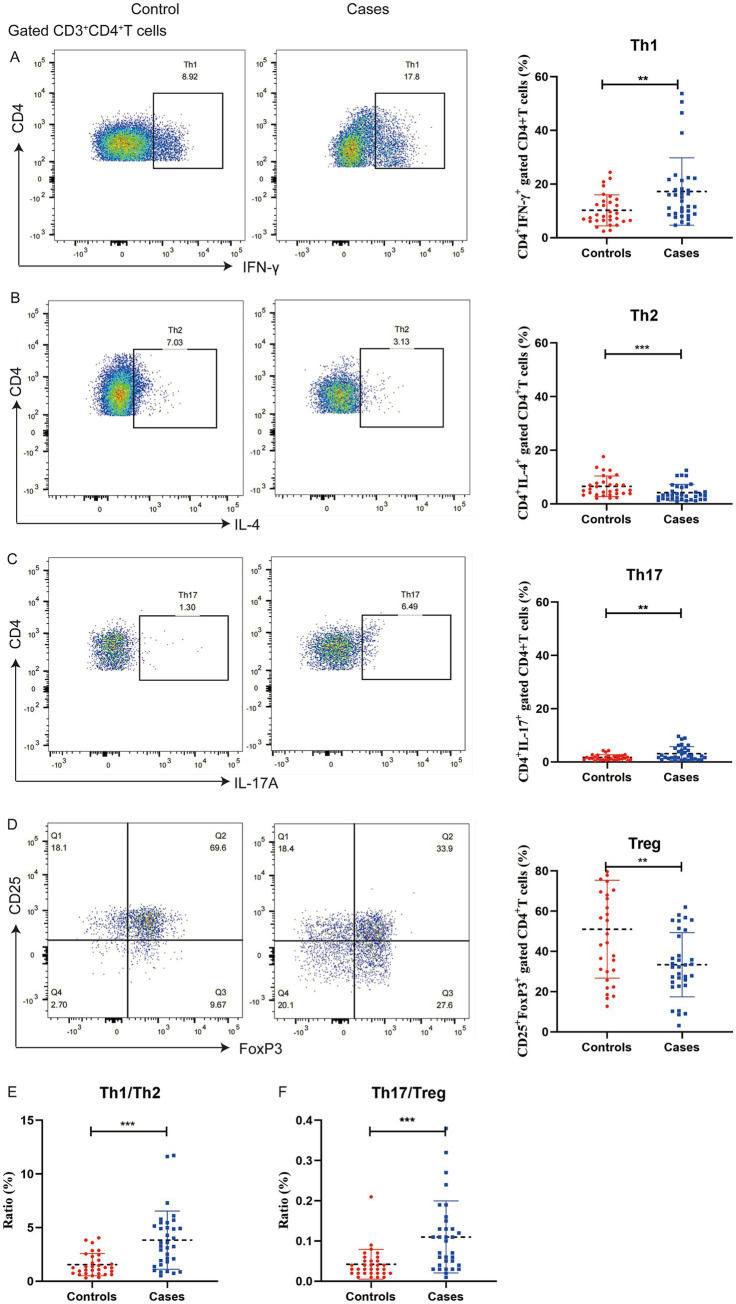
Distribution of T cell subsets in peripheral blood mononuclear cells from pregnant women with preeclampsia (*n* = 34) and without preeclampsia (*n* = 31). The percentages of IFN-γ^+^ of CD4^+^ T cells **(A)**, IL-4^+^ of CD4^+^ T cells **(B)**, IL-17A^+^ of CD4^+^ T cells **(C)**, and CD25^+^ FOXP3^+^ of CD4^+^ T cells **(D)** were determined using flow cytometry, the ratio of Th1/Th2 **(E)** and Th17/Treg **(F)**. ^**^p < 0.01 and ^***^p < 0.001.

The T cell populations in the two groups were presented in Figure 2. Women with preeclampsia had higher percentages of Th1 (17.22% versus 10.21%, *p* = 0.006) and Th17 cells (3.15% versus 1.69%, *p* = 0.004), and lower percentages of Th2 (4.16% versus 6.54%, *p* = 0.008) and Treg cells (33.39% versus 51.05%, *p* = 0.001) than controls. Additionally, the Th1/Th2 (3.83 versus 1.55, *p* < 0.001) and Th17/Treg ratios (0.11 versus 0.04, *p* < 0.001) were significantly elevated in women with preeclampsia.

The correlations of the vitamin D concentration with T cell subsets and the immune balance status are presented in Table 3. The vitamin D concentration was associated with Th1 cells, with correlation coefficients of *r* = −0.399 (*p* = 0.001), *r* = −0.282 (*p* = 0.023), and *r* = −0.339 (*p* = 0.006) between Th1 and 25(OH)D_2_, 25(OH)D_3_ as well as 25(OH)D, respectively. In the correlation analysis between vitamin D and immune balance of T cell subsets, only 25(OH)D_2_ was found to be associated with Th1/Th2 as well as Th17/Treg, the correlation coefficients for the associations 25(OH)D_2_ with the Th1/Th2 and Th17/Treg ratios were *r* = −0.321 (*p* = 0.009), and *r* = −0.245 (*p* = 0.049), respectively. The correlations between T cell subsets and preeclampsia were showed in Supplementary Table 6.

**Table 3:** Correlations between vitamin D forms and T cell subsets (*n* = 65).

T cell subsets	25(OH)D_2_		25(OH)D_3_		25(OH)D	
	*r*	*p*	*r*	*p*	*r*	*p*
CD4	0.176	0.164	0.186	0.142	0.187	0.139
Th1	**−0.399**	**0.001**	**−0.282**	**0.023**	**−0.339**	**0.006**
Th2	0.036	0.777	−0.195	0.120	−0.192	0.126
Th17	−0.097	0.442	−0.164	0.245	−0.096	0.449
Treg	0.237	0.057	0.073	0.565	0.105	0.464
Th1/Th2	**−0.321**	**0.009**	−0.046	0.718	−0.013	0.916
Th17/Treg	**−0.245**	**0.049**	−0.162	0.198	−0.204	0.103

The bold values indicate that the correlation between vitamin D forms and T cell subsets is statistically significant.

**Figure 3:**
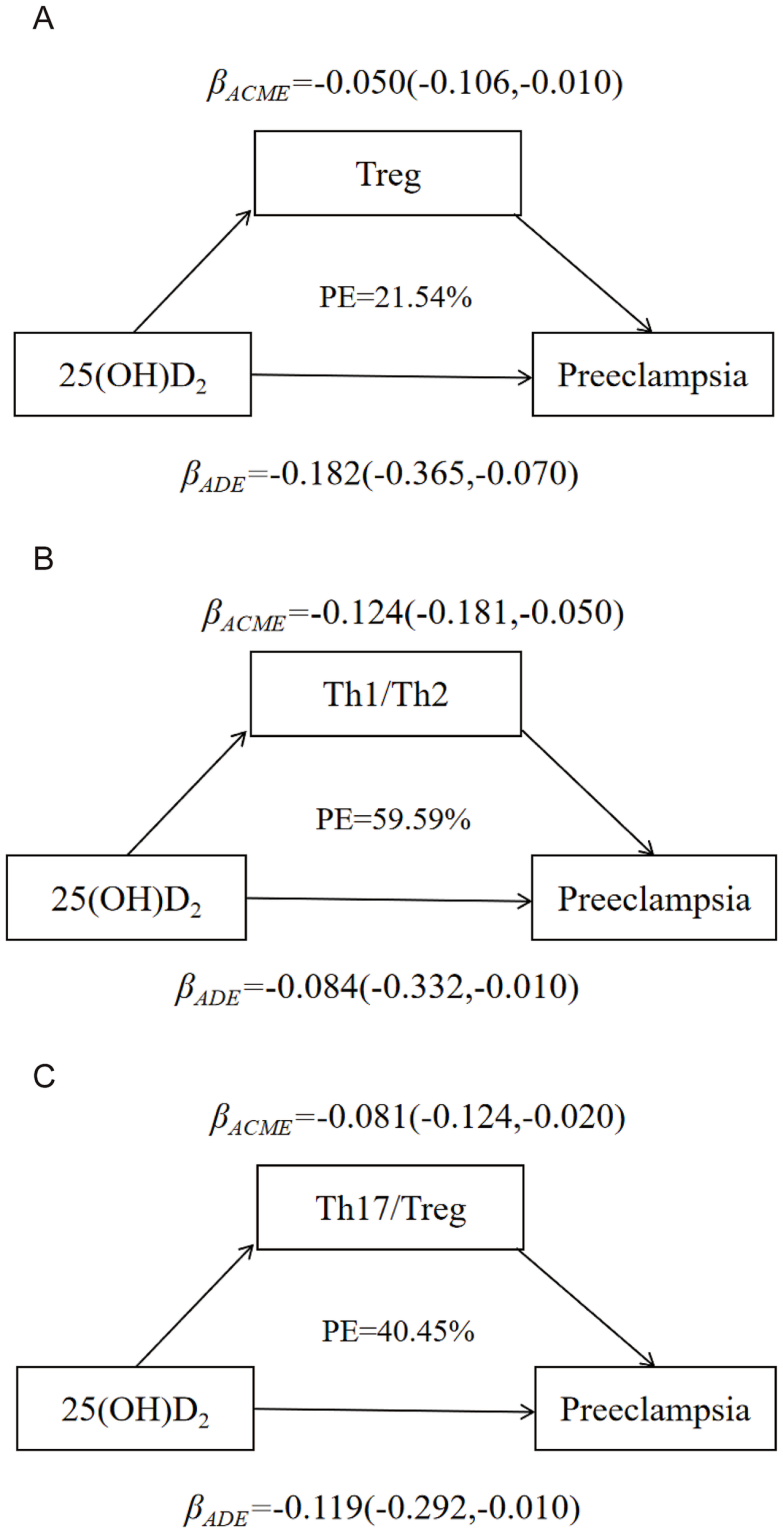
Model of the mediation effects of immune balance on the correlation between 25(OH)D_2_ and preeclampsia. **(A)** The mediating effect of Treg on the correlation between 25(OH)D_2_ and preeclampsia. **(B)** The mediating effect of Th1/Th2 balance on the correlation between 25(OH)D_2_ and preeclampsia. **(C)** The mediating effect of Th17/Treg balance on the correlation between 25(OH)D_2_ and preeclampsia. Maternal age, gestational week, parity (multiparous or primiparous), pre-pregnancy BMI, education level (high school or below, university or above) and monthly income (≤4,000 RMB or >4,000 RMB).

Figure 3 and Supplementary Tables 7–9 illustrated the model for the mediating effects of T cell subsets, Th1/Th2 as well as Th17/Treg ratios on the relationship between 25(OH)D_2_/25(OH)D_2_/25(OH)D and preeclampsia. The Th1/Th2 partly mediated the relationship between 25(OH)D_2_ and preeclampsia, with the significant direct effect of 25(OH)D_2_ on preeclampsia (*β*_direct_: −0.084, 95% CI: −0.332, −0.010) and significant indirect effect (*β*_indirect_: −0.124, 95%CI: −0.181, −0.050). The Th1/Th2 weakened the correlation between 25(OH)D_2_ and preeclampsia, and the respective mediation effect proportion was 59.59%, indicating that lower 25(OH)D_2_ could result in an imbalance between Th1 and Th2 and further increased the risk of preeclampsia. The similar mediation effects of Treg and Th17/Treg on 25(OH)D_2_ and preeclampsia were also found.

## Discussion

This case–control study found that women with preeclampsia had larger populations of Th1 and Th17 cells and lower levels of Th2 and Treg cells than the controls. Furthermore, Th1/Th2 and Th17/Treg ratios were negatively associated with the 25(OH)D_2_ concentration. The relationship between 25(OH)D_2_ and preeclampsia was weakened by the mediating effects of the Th1/Th2 and Th17/Treg ratio, a lower level of 25(OH)D_2_ may result in the imbalance between Th1/Th2 and Th17/Treg, thus increasing the risk of preeclampsia.

We previously found that a lower vitamin D concentration was associated with a higher risk of preeclampsia (OR: 0.26, 95% CI: 0.11, 0.60) (13). While, the mechanisms between vitamin D and preeclampsia still further exploration. Immune balance, particularly with respect to the Th1/Th2 and Th17/Treg balances, is considered an important immune mechanism underlying preeclampsia (12, 29). In a normal pregnancy, Th2 and Treg cells suppress the proliferation and activity of Th1 and Th17 cells to promote maternal-fetal tolerance and support a healthy pregnancy (30–33). However, in preeclampsia, the populations of Th1 and Th17 cells increase significantly, which disrupt maternal-fetal tolerance. These dominant of Th1 and Th17 cells further suppress the populations and functions of Th2 and Treg cells, exacerbating the immune imbalance of T cell subsets and contributing to the development of preeclampsia (29). Vitamin D plays a crucial role in modulating the immune system to promote a more tolerogenic immune environment by promoting Treg function and modulating T helper cell differentiation (17, 34, 35) and thus regulates the Th17/Treg and Th1/Th2 balance in women with infertility and recurrent pregnancy loss (36–38). While vitamin D deficiency can result in dominance of Th1 and Th17, as well as inhibition of the development and functions of Th2 and Treg cells, resulting in an imbalance between Th1 and Th2 as well as Th17 and Treg (39–41). Such imbalance contributes to immune system dysfunction and can lead to conditions such as hypertension, preeclampsia, and eclampsia. While, the mediating effects of Th1/Th2 and Th17/Treg ratios on vitamin D and preeclampsia were still unclear. In our study, we also observed that Th17/Treg and Th1/Th2 imbalances in women with preeclampsia. However, the concentration of cytokine in these two groups was not entirely consistent with those in previous studies or our flow cytometry results (40). This discrepancy may be attributable to various factors that influence cytokine levels, such as the gestational age, diet, and other environmental variables (40, 42).

Vitamin D exerts its biological effects by binding to vitamin D receptors on immune cells to promote Treg differentiation, enhance Th2 activity, and reduce Th17 and Th1 responses (43). It also has been shown to suppress the secretion of pro-inflammatory cytokines, such as TNF-α, IFN-γ, IL-2, and IL-6, in experimental models (34, 44, 45). The results of the current study suggest that 25(OH)D_2_ plays a key role in regulating the balance between Th1/Th2 and Th17/Treg balances and thereby influence the development of preeclampsia, as far as we know, this is the first study to explore the mediating effect of immune imbalance between 25(OH)D_2_ and preeclampsia. This effect may be due to the higher affinity of vitamin D receptors expressed by immune cells for 25(OH)D_2_ (46). This finding might help explain our findings that Th1/Th2 and Th17/Treg balances mediated the association between 25(OH)D_2_, but not 25(OH)D_3_, and preeclampsia.

### Strengths and limitations

The major strength of this study is that it is the first study to assess the mediating effect of Th1/Th2 as well as Th17/Treg imbalance on the relationship between vitamin D and preeclampsia in pregnant women. The results indicate that 25(OH)D_2_ is negatively associated with the Th1/Th2 and Th17/Treg immune balance, such that 25(OH)D_2_ deficiency increases the risk of preeclampsia by promoting Th1/Th2 and Th17/Treg immune imbalance. Second, we explored the mediating effects of Th1/Th2 as well as Th17/Treg immune imbalance on different forms of vitamin D and preeclampsia in this study. The results offer insights regarding the use of different forms of vitamin D to regulate immune balance in pregnant women and providing clues for regarding vitamin D_2_ or vitamin D_3_ supplementation to maintain immune balance and prevent preeclampsia.

However, some limitations of this study warrant further exploration. First, due to the case–control study design of our study, our findings just provide a supportive evidence instead of a direct causal evidence to support the mediating effect of Th1/Th2 and Th17/Treg balance on association between 25(OH)D_2_ and preeclampsia. Second, T cell subsets are more accurate and objective measure of immune status, while given its high cost and operational complexity, flow cytometry is unsuitable for large-scale studies, the relatively small sample size of T cell subsets from pregnant women may introduce potential residual confounding, highlighting the need for further replication in larger prospective studies to validate the findings obtained from this study.

## Conclusion

In summary, this study found inverse correlations between 25(OH)D_2_ and the Th1/Th2 and Th17/Treg ratios. Th1/Th2 and Th17/Treg imbalances were also found to mediate the relationship between the 25(OH)D_2_ concentration and risk of preeclampsia. Future prospective cohort studies and well-designed randomized controlled trials with larger sample of pregnant women are needed to further explore the mechanisms linking various forms of vitamin D, immune status and the risk of preeclampsia.

## Data Availability

The original contributions presented in the study are publicly available. This data can be found in Figshare: https://figshare.com/articles/dataset/Immune_imbalance_mediates_the_relationship_between_plasma_vitamin_D_concentration_and_preeclampsia_in_Chinese_pregnant_women_A_case-control_study/30464585.

## References

[1] Phipps EA Thadhani R Benzing T Karumanchi SA. Pre-eclampsia: pathogenesis, novel diagnostics and therapies. Nat Rev Nephrol. (2019) 15:275–89. doi: 10.1038/s41581-019-0119-6, PMID: 30792480 PMC6472952

[2] Abalos E Cuesta C Grosso AL Chou D Say L. Global and regional estimates of preeclampsia and eclampsia: a systematic review. Eur J Obstet Gynecol Reprod Biol. (2013) 170:1–7. doi: 10.1016/j.ejogrb.2013.05.005, PMID: 23746796

[3] American College of Obstetricians & Gynecologists. Hypertension in pregnancy. Report of the American College of Obstetricians and Gynecologists’ Task Force on hypertension in pregnancy. Obstet Gynecol. (2013):1122–31. doi: 10.1097/01.AOG.0000437382.03963.88 24150027

[4] Souza JP Gulmezoglu AM Vogel J Carroli G Lumbiganon P Qureshi Z . Moving beyond essential interventions for reduction of maternal mortality (the WHO multicountry survey on maternal and newborn health): a cross-sectional study. Lancet. (2013) 381:1747–55. doi: 10.1016/S0140-6736(13)60686-8, PMID: 23683641

[5] Epstein FH. Late vascular effects of toxemia of pregnancy. N Engl J Med. (1964) 271:391–5. doi: 10.1056/NEJM196408202710803, PMID: 14164656

[6] Mol BWJ Roberts CT Thangaratinam S Magee LA de Groot CJM Hofmeyr GJ. Pre-eclampsia. Lancet. (2016) 387:999–1011. doi: 10.1016/S0140-6736(15)00070-7, PMID: 26342729

[7] Strand KM Heimstad R Iversen AC Austgulen R Lydersen S Andersen GL . Mediators of the association between pre-eclampsia and cerebral palsy: population based cohort study. BMJ. (2013) 347:f4089. doi: 10.1136/bmj.f4089, PMID: 23838554 PMC3706637

[8] Hansen AR Barnes CM Folkman J McElrath TF. Maternal preeclampsia predicts the development of bronchopulmonary dysplasia. J Pediatr. (2010) 156:532–6. doi: 10.1016/j.jpeds.2009.10.018, PMID: 20004912

[9] Everett TR Wilkinson IB Lees CC. Drug development in preeclampsia: a ‘no go’ area? J Matern Fetal Neonatal Med. (2012) 25:50–2. doi: 10.3109/14767058.2011.557791, PMID: 21391756

[10] Meggyes M Miko E Lajko A Csiszar B Sandor B Matrai P . Involvement of the PD-1/PD-L1 co-inhibitory pathway in the pathogenesis of the inflammatory stage of early-onset preeclampsia. Int J Mol Sci. (2019) 20:583. doi: 10.3390/ijms20030583, PMID: 30700015 PMC6386834

[11] Jianjun Z Yali H Zhiqun W Mingming Z Xia Z. Imbalance of T-cell transcription factors contributes to the Th1 type immunity predominant in pre-eclampsia. Am J Reprod Immunol. (2010) 63:38–45. doi: 10.1111/j.1600-0897.2009.00763.x 19912158

[12] Ding H Dai Y Lei Y Wang Z Liu D Li R . Upregulation of CD81 in trophoblasts induces an imbalance of Treg/Th17 cells by promoting IL-6 expression in preeclampsia. Cell Mol Immunol. (2019) 16:302–12. doi: 10.1038/s41423-018-0186-9, PMID: 30487550 PMC6318306

[13] Huang XM Liu YH Zhang H Cao Y Dou WF Duan DD . Dietary and serum vitamin D and preeclampsia risk in Chinese pregnant women: a matched case–control study. Br J Nutr. (2021) 128:84–92. doi: 10.1017/S0007114521002956, PMID: 34353401

[14] Mirzakhani H Litonjua AA McElrath TF O’Connor G Lee-Parritz A Iverson R . Early pregnancy vitamin D status and risk of preeclampsia. J Clin Invest. (2016) 126:4702–15. doi: 10.1172/JCI89031, PMID: 27841759 PMC5127689

[15] van der Pligt P Willcox J Szymlek-Gay EA Murray E Worsley A Daly RM. Associations of maternal vitamin D deficiency with pregnancy and neonatal complications in developing countries: a systematic review. Nutrients. (2018) 10:640. doi: 10.3390/nu10050640, PMID: 29783717 PMC5986519

[16] Achkar M Dodds L Giguere Y Forest JC Armson BA Woolcott C . Vitamin D status in early pregnancy and risk of preeclampsia. Am J Obstet Gynecol. (2015) 212:511.e1–7. doi: 10.1016/j.ajog.2014.11.009, PMID: 25446694 PMC5023419

[17] Zheng S Dong S Shen H Xu P Shu C. Role of vitamin D in the pathogenesis of early-onset preeclampsia: a narrative review. Front Nutr. (2025) 12:1598691. doi: 10.3389/fnut.2025.1598691, PMID: 40607031 PMC12213361

[18] Hypertensive Disorders in Pregnancy Subgroup, Chinese Society of Obstetrics and Gynecology, Chinese Medical Association. Diagnosis and treatment guideline of hypertensive disorders in pregnancy (2015). Zhonghua Fu Chan Ke Za Zhi. (2015) 50:721–8. doi: 10.3760/cma.j.issn.0529-567x.2015.10.001 26675569

[19] Cao Y Liu Y Zhao X Duan D Dou W Fu W . Adherence to a dietary approaches to stop hypertension (DASH)-style diet in relation to preeclampsia: a case–control study. Sci Rep. (2020) 10:9078. doi: 10.1038/s41598-020-65912-2, PMID: 32493995 PMC7270088

[20] Zeng G Zhang G Chen X. Th1 cytokines, true functional signatures for protective immunity against TB? Cell Mol Immunol. (2018) 15:206–15. doi: 10.1038/cmi.2017.113, PMID: 29151578 PMC5843617

[21] Nakayama T Hirahara K Onodera A Endo Y Hosokawa H Shinoda K . Th2 cells in health and disease. Annu Rev Immunol. (2017) 35:53–84. doi: 10.1146/annurev-immunol-051116-052350, PMID: 27912316

[22] Neurath MF Finotto S Glimcher LH. The role of Th1/Th2 polarization in mucosal immunity. Nat Med. (2002) 8:567–73. doi: 10.1038/nm0602-567, PMID: 12042806

[23] Field CS Baixauli F Kyle RL Puleston DJ Cameron AM Sanin DE . Mitochondrial integrity regulated by lipid metabolism is a cell-intrinsic checkpoint for Treg suppressive function. Cell Metab. (2020) 31:422–37 e5. doi: 10.1016/j.cmet.2019.11.021 31883840 PMC7001036

[24] Summers SA Steinmetz OM Li M Kausman JY Semple T Edgtton KL . Th1 and Th17 cells induce proliferative glomerulonephritis. J Am Soc Nephrol. (2009) 20:2518–24. doi: 10.1681/ASN.2009030337, PMID: 19820122 PMC2794236

[25] Ghoreschi K Thomas P Breit S Dugas M Mailhammer R van Eden W . Interleukin-4 therapy of psoriasis induces Th2 responses and improves human autoimmune disease. Nat Med. (2003) 9:40–6. doi: 10.1038/nm804, PMID: 12461524

[26] Barbi J Pardoll D Pan F. Treg functional stability and its responsiveness to the microenvironment. Immunol Rev. (2014) 259:115–39. doi: 10.1111/imr.12172, PMID: 24712463 PMC3996455

[27] Zhang Y Liu Z Tian M Hu X Wang L Ji J . The altered PD-1/PD-L1 pathway delivers the ‘one-two punch’ effects to promote the Treg/Th17 imbalance in pre-eclampsia. Cell Mol Immunol. (2018) 15:710–23. doi: 10.1038/cmi.2017.70, PMID: 28890543 PMC6123412

[28] Wang S Li M Lin H Wang G Xu Y Zhao X . Amino acids, microbiota-related metabolites, and the risk of incident diabetes among normoglycemic Chinese adults: findings from the 4C study. Cell Rep Med. (2022) 3:100727. doi: 10.1016/j.xcrm.2022.100727, PMID: 35998626 PMC9512668

[29] Darmochwal-Kolarz D Kludka-Sternik M Tabarkiewicz J Kolarz B Rolinski J Leszczynska-Gorzelak B . The predominance of Th17 lymphocytes and decreased number and function of Treg cells in preeclampsia. J Reprod Immunol. (2012) 93:75–81. doi: 10.1016/j.jri.2012.01.006, PMID: 22370101

[30] Lin H Mosmann TR Guilbert L Tuntipopipat S Wegmann TG. Synthesis of T helper 2-type cytokines at the maternal-fetal interface. J Immunol. (1993) 151:4562–73. 8409418

[31] Wang W Sung N Gilman-Sachs A Kwak-Kim J. T helper (Th) cell profiles in pregnancy and recurrent pregnancy losses: Th1/Th2/Th9/Th17/Th22/Tfh cells. Front Immunol. (2020) 11:2025. doi: 10.3389/fimmu.2020.02025, PMID: 32973809 PMC7461801

[32] Robertson SA Care AS Moldenhauer LM. Regulatory T cells in embryo implantation and the immune response to pregnancy. J Clin Invest. (2018) 128:4224–35. doi: 10.1172/JCI122182, PMID: 30272581 PMC6159994

[33] Robertson SA Green ES Care AS Moldenhauer LM Prins JR Hull ML . Therapeutic potential of regulatory T cells in preeclampsia-opportunities and challenges. Front Immunol. (2019) 10:478. doi: 10.3389/fimmu.2019.00478, PMID: 30984163 PMC6448013

[34] Sassi F Tamone C D’Amelio P. Vitamin D: nutrient, hormone, and immunomodulator. Nutrients. (2018) 10:1656. doi: 10.3390/nu10111656 30400332 PMC6266123

[35] Prietl B Treiber G Pieber TR Amrein K. Vitamin D and immune function. Nutrients. (2013) 5:2502–21. doi: 10.3390/nu5072502, PMID: 23857223 PMC3738984

[36] Ikemoto Y Kuroda K Nakagawa K Ochiai A Ozaki R Murakami K . Vitamin D regulates maternal T-helper cytokine production in infertile women. Nutrients. (2018) 10:902. doi: 10.3390/nu10070902, PMID: 30011861 PMC6073370

[37] Rafiee M Gharagozloo M Ghahiri A Mehrabian F Maracy MR Kouhpayeh S . Altered Th17/Treg ratio in recurrent miscarriage after treatment with paternal lymphocytes and vitamin D3: a double-blind placebo-controlled study. Iran J Immunol. (2015) 12:252–62. 26714417 10.22034/iji.2015.16754

[38] Ji J Zhai H Zhou H Song S Mor G Liao A. The role and mechanism of vitamin D-mediated regulation of Treg/Th17 balance in recurrent pregnancy loss. Am J Reprod Immunol. (2019) 81:e13112. doi: 10.1111/aji.13112, PMID: 30903715

[39] Granger JP. Inflammatory cytokines, vascular function, and hypertension. Am J Physiol Regul Integr Comp Physiol. (2004) 286:R989–90. doi: 10.1152/ajpregu.00157.2004, PMID: 15142853

[40] Aggarwal R Jain AK Mittal P Kohli M Jawanjal P Rath G. Association of pro- and anti-inflammatory cytokines in preeclampsia. J Clin Lab Anal. (2019) 33:e22834. doi: 10.1002/jcla.22834, PMID: 30666720 PMC6528584

[41] Jokhi PP King A Loke YW. Cytokine production and cytokine receptor expression by cells of the human first trimester placental-uterine interface. Cytokine. (1997) 9:126–37. doi: 10.1006/cyto.1996.0146, PMID: 9071564

[42] Rodriguez-Santana Y Ochoa JJ Lara-Villoslada F Kajarabille N Saavedra-Santana P Hurtado JA . Cytokine distribution in mothers and breastfed children after omega-3 LCPUFAs supplementation during the last trimester of pregnancy and the lactation period: a randomized, controlled trial. Prostaglandins Leukot Essent Fatty Acids. (2017) 126:32–8. doi: 10.1016/j.plefa.2017.09.006, PMID: 29031393

[43] Yang CY Leung PS Adamopoulos IE Gershwin ME. The implication of vitamin D and autoimmunity: a comprehensive review. Clin Rev Allergy Immunol. (2013) 45:217–26. doi: 10.1007/s12016-013-8361-3, PMID: 23359064 PMC6047889

[44] Carvalho JTG Schneider M Cuppari L Grabulosa CC D TA Qr BM . Cholecalciferol decreases inflammation and improves vitamin D regulatory enzymes in lymphocytes in the uremic environment: a randomized controlled pilot trial. PLoS One. (2017) 12:e0179540. doi: 10.1371/journal.pone.0179540 28665937 PMC5493305

[45] Xie Z Chen J Zheng C Wu J Cheng Y Zhu S . 1,25-dihydroxyvitamin D3 -induced dendritic cells suppress experimental autoimmune encephalomyelitis by increasing proportions of the regulatory lymphocytes and reducing T helper type 1 and type 17 cells. Immunology. (2017) 152:414–24. doi: 10.1111/imm.12776, PMID: 28617989 PMC5629429

[46] Chun RF Peercy BE Orwoll ES Nielson CM Adams JS Hewison M. Vitamin D and DBP: the free hormone hypothesis revisited. J Steroid Biochem Mol Biol. (2014) 144:132–7. doi: 10.1016/j.jsbmb.2013.09.012, PMID: 24095930 PMC3976473

