# Supplementary material for: Immune imbalance mediates the relationship between plasma vitamin D concentration and preeclampsia in Chinese pregnant women: a case–control study
Source: Front Nutr. 2025 Nov 14;12:1665593. doi: 10.3389/fnut.2025.1665593 (PMC12661851; doi:10.3389/fnut.2025.1665593)
Supplement: Supplementary file 1 [file Table_1.DOCX]

**Supplementary**

**Supplementary Table 1**

The distribution of 65 flow cytometry samples between cases and controls

| Variables | Cases (n=34) | Controls (n=31) | *P* |
| --- | --- | --- | --- |
| Maternal age (years) | 30.76±5.20 | 30.26±3.41 | 0.647 |
| Gestational week (weeks) | 33.76±2.74 | 34.03±2.29 | 0.672 |
| Parity |  |  | 0.405 |
| Multiparous | 17(51.52） | 11(40.74) |  |
| Primiparous | 16(48.48) | 16(59.26) |  |
| SBP (mmHg) | 148.94±16.31 | 109.44±8.40 | ＜0.001 |
| DBP (mmHg) | 97.06±12.99 | 71.56±9.10 | ＜0.001 |
| Pre-pregnancy BMI (kg/m^2^) | 23.56±3.33 | 21.97±2.85 | 0.055 |
| Weight gain (kg) | 16.43±5.80 | 12.30±5.08 | 0.006 |
| Monthly income |  |  | 0.200 |
| ≤4000RMB | 19(65.52) | 11(47.83) |  |
| >4000RMB | 10(34.48) | 12(52.17) |  |
| Education level |  |  | 0.525 |
| High school or below | 21(63.64) | 15(55.56) |  |
| University or above | 12(36.36) | 12(44.44) |  |
| GDM |  |  | 0.414 |
| No | 29(85.29) | 24(77.42) |  |
| Yes | 5(14.71) | 7(22.58) |  |
| 25(OH)D_2_ (ng/mL) | 0.78(0.61,1.22) | 0.98(0.92,1.71) | 0.009 |
| 25(OH)D_3_ (ng/mL) | 11.45(7.42,17.20) | 11.40(9.19,20.80) | 0.400 |
| 25(OH)D (ng/mL) | 12.60(8.00,18.40) | 13.40(10.50,22.40) | 0.235 |

DBP: diastolic blood pressure; SBP: systolic blood pressure; BMI: Body Mass Index; GDM: gestational diabetes

**Supplementary Table 2**

Correlations between cytokines and preeclampsia (n=373)

| Variables | Preeclampsia status | |
| --- | --- | --- |
|  | *r* | *P* |
| IFN-γ | 0.114 | 0.028 |
| IL-4 | 0.157 | 0.002 |
| IL-17A | 0.116 | 0.027 |
| IL-10 | 0.014 | 0.793 |
| IFN-γ/IL-4 | -0.034 | 0.528 |
| Th17/IL-10 | 0.066 | 0.219 |

**Supplementary Table 3**

Mediation analysis of immune imbalance (based on ELISA) in the relationship between 25(OH)D_2_ and preeclampsia

| Variables | Total effect  *β*（95%*CI*） | Direct effect  *β*（95%*CI*） | Indirect effect  *β*（95%*CI*） | PE  (%) |
| --- | --- | --- | --- | --- |
| IFN-γ | -0.202(-0.283,-0.140) | -0.204(-0.286,-0.140) | 0.002(-0.003,0.010) | 0.94 |
| IL-4 | -0.212(-0.289,-0.150) | -0.208(-0.286,-0.150) | -0.004(-0.010,0.000) | 1.69 |
| IL-17A | -0.206(-0.286,-0.140) | -0.203(-0.280,-0.140) | -0.003(-0.009,0.000) | 1.59 |
| IL-10 | -0.214(-0.293,-0.150) | -0.215(-0.295,-0.150) | 0.001(-0.001,0.010) | 0.29 |
| IFN-γ/IL-4 | -0.201(-0.283,-0.140) | -0.200(-0.284,-0.140) | -0.001(-0.005,0.001) | 0.30 |
| Th17/IL-10 | -0.213(-0.291,-0.150) | -0.210(-0.291,-0.150) | -0.002(-0.006,0.000) | 0.10 |

Maternal age, gestational week, parity (multiparous or primiparous), pre-pregnancy BMI, education level (high school or below, university or above) and monthly income ( ≤4000RMB or > 4000RMB) were adjusted in mediating model.

**Supplementary Table 4**

Mediation analysis of immune imbalance (based on ELISA) in the relationship between 25(OH)D_3_ and preeclampsia

| Variables | Total effect  *β*（95%*CI*） | Direct effect  *β*（95%*CI*） | Indirect effect  *β*（95%*CI*） | PE  (%) |
| --- | --- | --- | --- | --- |
| IFN-γ | -0.011(-0.017,-0.010) | -0.011(-0.017,-0.010) | -0.000(-0.001,0.000) | 1.79 |
| IL-4 | -0.012(-0.017,-0.010) | -0.012(-0.018,-0.010) | 0.000(-0.001,0.000) | 1.00 |
| IL-17A | -0.011(-0.016,-0.010) | -0.011(-0.016,0.000) | -0.001(-0.002,0.000) | 4.87 |
| IL-10 | -0.011(-0.017,0.000) | -0.011(-0.017,0.000) | 0.000(0.000,0.000) | 0.01 |
| IFN-γ/IL-4 | -0.012(-0.017,-0.010) | -0.012(-0.017,-0.010) | 0.000(-0.001,0.000) | 0.84 |
| Th17/IL-10 | -0.011(-0.017,0.000) | -0.011(-0.017,0.000) | 0.000(-0.001,0.000) | 1.95 |

Maternal age, gestational week, parity (multiparous or primiparous), pre-pregnancy BMI, education level (high school or below, university or above) and monthly income ( ≤4000RMB or > 4000RMB) were adjusted in mediating model.

**Supplementary Table 5**

Mediation analysis of immune imbalance (based on ELISA) in the relationship between 25(OH)D and preeclampsia

| Variables | Total effect  *β*（95%*CI*） | Direct effect  *β*（95%*CI*） | Indirect effect  *β*（95%*CI*） | PE  (%) |
| --- | --- | --- | --- | --- |
| IFN-γ | -0.013(-0.018,-0.010) | -0.013(-0.018,-0.010) | 0.000(-0.001,0.000) | 1.40 |
| IL-4 | -0.014(-0.017,-0.010) | -0.013(-0.018,-0.010) | 0.000(-0.001,0.000) | 1.27 |
| IL-17A | -0.013(-0.017,-0.010) | -0.012(-0.016,-0.010) | 0.000(-0.001,0.000) | 4.0 |
| IL-10 | -0.013(-0.018,-0.010) | -0.013(-0.018,-0.010) | 0.000(0.001,0.000) | 0.05 |
| IFN-γ/IL-4 | -0.013(-0.017,-0.010) | -0.013(-0.017,-0.010) | 0.000(0.000,0.000) | 0.07 |
| Th17/IL-10 | -0.013(-0.017,-0.010) | -0.013(-0.017,-0.010) | 0.000(-0.001,0.000) | 1.48 |

Maternal age, gestational week, parity (multiparous or primiparous), pre-pregnancy BMI, education level (high school or below, university or above) and monthly income ( ≤4000RMB or > 4000RMB) were adjusted in mediating model.

**Supplementary Table 6**

Correlations between T cell subsets and preeclampsia (n=65)

| Variables | Preeclampsia status | |
| --- | --- | --- |
|  | *r* | *P* |
| CD4 | 0.006 | 0.961 |
| Th1 | 0.293 | 0.018 |
| Th2 | -0.361 | 0.003 |
| Th17 | 0.347 | 0.005 |
| Treg | -0.407 | 0.001 |
| Th1/Th2 | 0.531 | <0.001 |
| Th17/Treg | 0.441 | <0.001 |

**Supplementary Table 7**

Mediation analysis of immune imbalance (based on flowmetry) in the relationship between 25(OH)D_2_ and preeclampsia

| Variables | Total effect  *β*（95%*CI*） | Direct effect  *β*（95%*CI*） | Indirect effect  *β*（95%*CI*） | PE  (%) |
| --- | --- | --- | --- | --- |
| Th1 | -0.226(-0.412, -0.13) | -0.203(-0.409, -0.080) | -0.023(-0.084,0.020) | 9.99 |
| Th2 | -0.253(-0.484,-0.130) | -0.231(-0.453,-0.110) | -0.022(-0.082,0.030) | 8.86 |
| Th17 | -0.232(-0.405,-0.080) | -0.202(-0.392,-0.070) | -0.030(-0.072,0.010) | 12.98 |
| Treg | -0.232(-0.402,-0.130) | -0.182(-0.365,-0.070) | -0.050(-0.106,-0.010) | **21.54** |
| Th1/Th2 | -0.208(-0.406,-0.080) | -0.084(-0.332,-0.010) | -0.124(-0.181,-0.050) | **59.59** |
| Th17/Treg | -0.120(-0.342,-0.070) | -0.119(-0.292,-0.010) | -0.081(-0.124,-0.020) | **40.45** |

Maternal age, gestational week, parity (multiparous or primiparous), pre-pregnancy BMI, education level (high school or below, university or above) and monthly income ( ≤4000RMB or > 4000RMB) were adjusted in mediating model.

**Supplementary Table 8**

Mediation analysis of immune imbalance (based on flowmetry) in the relationship between 25(OH)D_3_ and preeclampsia

| Variables | Total effect  *β*（95%*CI*） | Direct effect  *β*（95%*CI*） | Indirect effect  *β*（95%*CI*） | PE  (%) |
| --- | --- | --- | --- | --- |
| Th1 | -0.012(-0.020,0.010) | -0.007(-0.018,0.010) | -0.005(-0.011,0.000) | 40.12 |
| Th2 | -0.011(-0.021,0.010) | -0.017(-0.025,0.000) | -0.056(-0.001,0.010) | 49.14 |
| Th17 | -0.011(-0.019,0.010) | -0.008(-0.018,0.010) | -0.003(-0.008,0.000) | 25.45- |
| Treg | -0.011(-0.020,0.010) | -0.007(-0.016,0.010) | -0.004(-0.009,0.000) | 36.96 |
| Th1/Th2 | -0.011(-0.022,0.010) | -0.013(-0.026,0.000) | 0.002(-0.006,0.010) | 16.85 |
| Th17/Treg | -0.012(-0.019,0.000) | -0.001(-0.010,0.010) | -0.011(-0.017,0.000) | 89.05 |

Maternal age, gestational week, parity (multiparous or primiparous), pre-pregnancy BMI, education level (high school or below, university or above) and monthly income ( ≤4000RMB or > 4000RMB) were adjusted in mediating model.

**Supplementary Table 9**

Mediation analysis of immune imbalance (based on flowmetry) in the relationship between 25(OH)D and preeclampsia

| Variables | Total effect  *β*（95%*CI*） | Direct effect  *β*（95%*CI*） | Indirect effect  *β*（95%*CI*） | PE  (%) |
| --- | --- | --- | --- | --- |
| Th1 | -0.013(-0.018,0.000) | -0.009(-0.017,0.010) | -0.004(-0.010,0.000) | 33.20 |
| Th2 | -0.013(-0.020,0.000) | -0.017(-0.025,-0.010) | 0.004(-0.002,0.010) | 34.37 |
| Th17 | -0.013(-0.018,0.000) | -0.010(-0.017,0.000) | -0.003(-0.008,0.000) | 22.74 |
| Treg | -0.012(-0.019,0.000) | -0.008(-0.015,0.000) | -0.004(-0.009,0.000) | 33.92 |
| Th1/Th2 | -0.013(-0.021,0.010) | -0.013(-0.023,0.000) | -0.001(-0.001,0.010) | 1.90 |
| Th17/Treg | -0.012(-0.018,0.000) | -0.002(-0.010,0.010) | -0.010(-0.015,0.000) | 80.34 |

Maternal age, gestational week, parity (multiparous or primiparous), pre-pregnancy BMI, education level (high school or below, university or above) and monthly income ( ≤4000RMB or > 4000RMB) were adjusted in mediating model.

# 
